# The elastic properties of the tendinous and capsular layers of the rotator cuff complex using fresh tissue—a biomechanical study

**DOI:** 10.1007/s00590-024-04168-2

**Published:** 2025-02-11

**Authors:** Jessica Y. Cronje, Nkhensani Mogale, Shavana Govender, Mathys A. de Beer, Abrie J. Oberholster, Chris McDuling, Rudi Verbeek, Tshifhiwa Nkwenika, Natalie Keough

**Affiliations:** 1 University of Pretoria, Pretoria, South Africa; 2 University of the Witwatersrand, Johannesburg, South Africa; 3 Life Groenkloof Hospital, Pretoria, South Africa; 4 Council for Scientific and Industrial Research, Pretoria, South Africa; 5 Elite Surgical, Pretoria, South Africa; 6 South African Medical Research Council, Pretoria, South Africa; 7 University of Warwick, Coventry, UK

**Keywords:** Supraspinatus, Infraspinatus, Subscapularis, Tendinous layer, Capsular layer, Elastic modulus

## Abstract

**Background:**

Elastic modulus is an important biomechanical component that indicates stiffness or elasticity of biological material. Recently the use of digital image correlation (DIC) in elastic modulus studies on fresh tissue has shown great accuracy in estimating elastic properties; thus, the aim of this study was to investigate the elasticity of capsular and tendinous layers of the rotator cuff complex employing this method.

**Materials and methods:**

The supraspinatus, infraspinatus, and subscapularis from eight (n = 8) fresh/frozen tissue shoulders were reverse dissected from their origins. The muscles were separated from one another and dissected to produce 20 × 20 mm tendinous and capsular strips for each muscle. DIC was employed to measure the strain of the tendinous and capsular portions of each of the muscles during tensile testing, and tangent elastic modulus values were obtained.

**Results:**

The tendinous layers for supraspinatus, infraspinatus, and subscapularis yielded higher average tangent elastic moduli readings (62.1 MPa, 67.1 MPa, and 59.6 MPa, respectively) compared to their capsular counterparts (29.0 MPa, 32.5 MPa, and 41.5 MPa, respectively).

**Conclusion:**

Different elastic moduli findings for the tendinous and capsular layers suggest these layers should be considered independently during surgical repair to avoid biomechanical imbalance which may result if these layers were to be repaired as one singular layer.

## Introduction

The efficacy of rotator cuff (RC) repair and management following pathologic or disease alteration greatly depends on the surgical or conservative methods employed. These methods in turn rely not only on the preference and experience of the surgeon or physician but also a thorough understanding and knowledge of the anatomical complexities of this structure. Biomechanical function and properties of the components that make up the RC unit form an intrinsic part of this anatomical complexity.

The RC has been well established in recent literature as an interdigitated complex inserting across the humeral tuberosities rather than, as previously described, distinct tendons inserting at individual locations [1–3]. The RC has two macroscopic and five histologic layers. The histology described by Clark and Harryman is as follows: the first layer consists of superficial fibres of the coracohumeral ligament and large arterioles; the second layer contains closely packed, parallel tendon fibres, with arteriole contributions from the first layer; the third layer is composed of smaller, less uniformly oriented fascicles and is thick and tendinous with smaller arteries; the fourth layer comprises loose connective tissue with thick collagen fibre bands that run perpendicular to the main cuff fibres, with only capillaries; finally, the fifth, deepest layer contains the most randomly organized fibres, consisting of a continuous sheet of interwoven collagen fibrils [1]. These histologically distinct regions of the RC muscles translate as macroscopic tendinous (consisting mainly of histological layer two to three) and capsular (mainly considered histological layer five) layers [1, 2, 4, 5]. The macroscopic layers can be easily visualized towards their insertion, where it was noted especially the third histologic layer splays, with marked interdigitation, as the muscle nears the humeral tuberosities [1].

Research on RC tear site prevalence notes articular-sided (affecting the capsular layer) tears are more common than bursal (tendinous)-sided tears [6–9]. Operative interventions for these tears have shown more favourable outcomes on follow-up postoperatively for articular repairs. Interestingly, bursal-sided tears have shown greater post-operative re-tear percentage [10–13]. Research on reconstructed RC tissue has shown that an inflammatory infiltration response may contribute to re-tearing and the use of anti-inflammatory medication and more recently extracellular vesicles to promote regeneration has shown success in these and other tissues [14, 15].

The aetiology of RC tears being multifactorial has both extrinsically (acromion sloping, coracoacromial ligament, acromioclavicular joint spurs etc.) and intrinsically (age, avascularity, biomechanical properties) documented causes. The greater prevalence of articular compared to bursal-sided tears has been linked to the intrinsic histological regions and their biomechanical properties [3]. One such biomechanical property is elastic modulus, which is a measure of the resistance of a material to deform when load is applied [16].

Awareness of biomechanical complexities has, in the age of 3D modelling, led to construction of various finite element models to investigate efficacy of various reparative techniques targeting different presentations of RC tears and gain a deeper understanding of the complex as a whole, as well as the pathophysiology that can occur within [17–19]. However, the simplicity of these models in terms of consideration for the macroscopically unique layers, as well as the paucity of studies on mechanical differences in the infraspinatus and subscapularis specifically, complicates the value and validation of results drawn from these models.

Recent literature highlights the use of DIC analysis as being most precise in measuring strain biological soft tissues undergo during tensile loading, from which elastic modulus values can be obtained [20–24]. Using DIC produces a more accurate data yield associated with elastic modulus pertaining to soft tissue samples, compared to using traditional grip-to-grip displacement of tensile testing machines which inherently include compliance effects at the clamp/specimen interface. The present study thus used the TEMA motion analysis software’s DIC algorithm to provide a more detailed description and create a better understanding of how the tendinous and capsular layers of the supraspinatus, infraspinatus, and subscapularis differ biomechanically in terms of elasticity.

## Materials and methods

Eight (n = 8) fresh/frozen human shoulders were obtained from the National Tissue Bank (Ethics Committee approval number 384/2018). The sample consisted of male and female shoulders equally divided between left and right, with an age range of 44–88 years. Where pathology was noted, the affected portion of the RC was excluded from testing. Sex, age, and population did not constitute exclusion criteria.

**Fig. 1:**
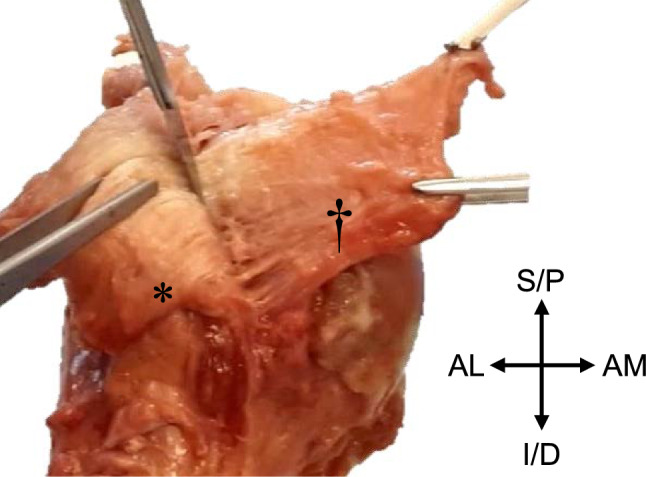
Proximal humerus showing sharp dissection through the fascial plane connecting the tendinous (*) and capsular (†) layers of the subscapularis of a right arm. AL—anterolateral; AM—anteromedial; I/D—inferior/distal; S/P—superior/proximal

The skin, fat, translucent fascia, and all scapular muscles, except supraspinatus, infraspinatus, and subscapularis, were removed from the scapula, clavicle, and humerus. An electric bone saw was utilized to saw through the humeral shaft approximately 200 mm from the apex of the head of the humerus. The supraspinatus, infraspinatus, and subscapularis were reverse dissected away from the scapula towards their insertion on the humerus and sectioned into portions of 20 × 20 mm wide strips. Upon sharp dissection of each section 20 mm from the humeral insertion, the tendinous and capsular layers could be visualized as individual layers, connected through a fascial plane, in accordance with previous literature [1]. This fascial plane was clearly visible once the strips had been cut into their 20 × 20 mm segments and the superficial tendinous and deeper capsular components simultaneously ‘pulled.’ These layers were then carefully dissected away from one another (Fig. 1). The dissection yielded 6 strips from each of the 8 shoulders as follows: supraspinatus capsular (SSC) and tendinous (SST), infraspinatus capsular (ISC) and tendinous (IST), subscapularis capsular (SCC) and tendinous (SCT) components. Signs of degeneration and/pathology led to the exclusion of seven RC strips, thus resulting in a final 41 (n = 41) of the original 48 strips being included for testing. Each strip was measured using a digital sliding calliper.

**Fig. 2:**
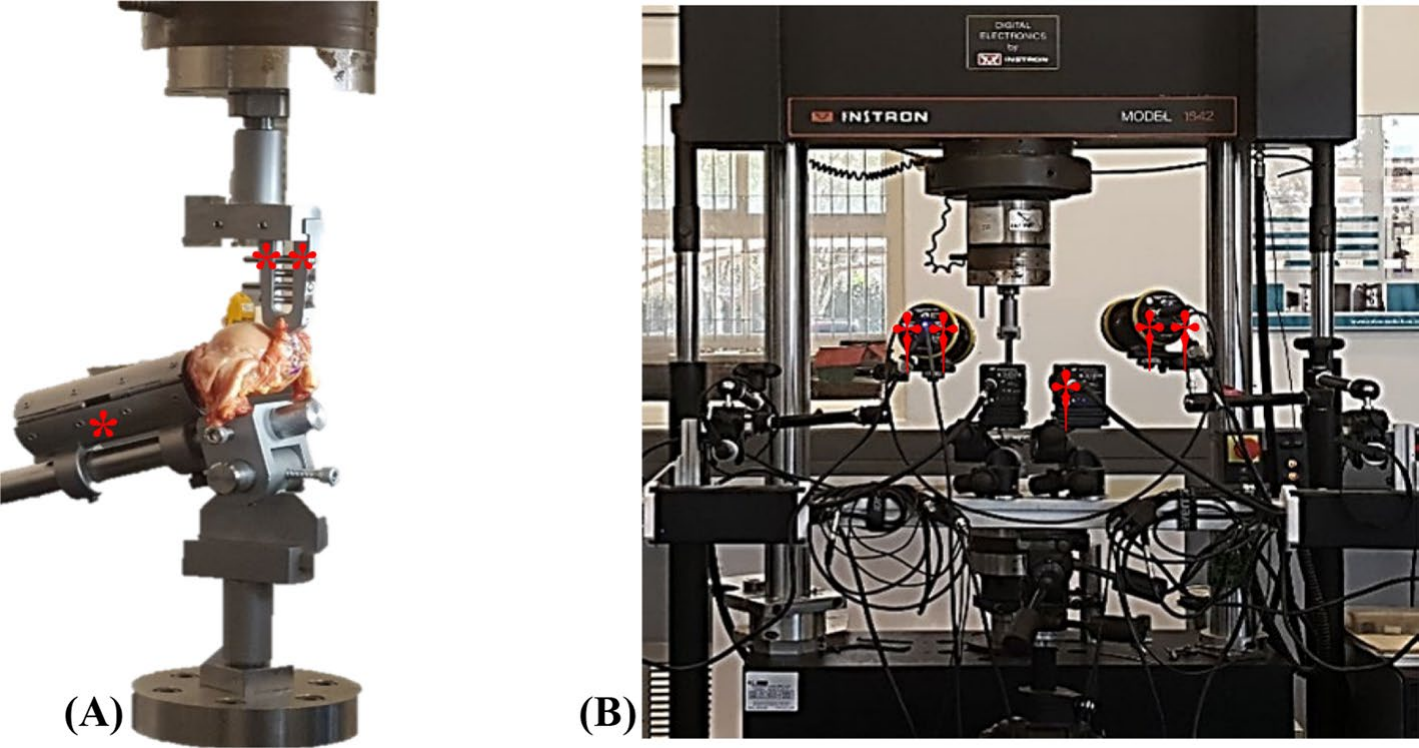
**A** Humeral shaft clamped (*) and mounted on the fixed base plate, with the tendinous strip of the supraspinatus clamped (**) and mounted to the load cell. **B** DIC camera (†) mounted on a stand and aimed at the testing area of the Instron with two accompanying lights (††)

Testing of each strip was done by securing the humeral shaft in a clamp designed to be mounted to the fixed base plate and clamping one of the six strips (enclosed in sandpaper to reinforce grip and reduce artificial tearing) in a separate clamp secured to the load cell of the Instron (25kN servo-hydraulic universal testing machine, model 1342) as seen in Fig. 2(A). Standard phosphate-buffered saline solution was applied to the strips throughout testing to prevent tissue desiccation. The anatomical complexity of the RC muscles to stabilize the glenohumeral joint needed careful consideration when clamping the individual segments during testing. Each strip was clamped in a position that allowed for orientation where the intact insertional end of the strip attached to the base plate was clamped in an orientation closely resembling how the individual segment would originate from the scapula. This clamping was kept consistent between specimens to ensure the results were obtained for similar loading conditions. Clamping in a position resembling anatomical directionality of the humerus and RC segments was occasionally restricted by testing setup and equipment, including clamp size.

To observe the range of elasticity each strip presented individually, an IDT NX8-S2 camera was set up, capturing tensile testing images for DIC analysis. A 50-mm lens was used at approximately 435 mm from the testing area, obtaining a field view of 120 × 90 mm as seen in Fig. 2(B). To eliminate lens distortion and calculate camera orientation, the camera setup was calibrated using a square-shaped pattern calibration panel with a pitch distance of 7 mm. The yield of this setup was a ten-frame-per-second single-camera capturing of the speckle pattern deformation in the RC strip analysed. A CoCo-80X digital signal analyser additionally recorded the displacement and load measurements taken by the Instron.

Because DIC is a non-contact, full-field measurement technique, it is ideal for measuring strain on soft tissue. DIC requires the following to yield useful data: (1) the speckle pattern should be sufficiently random and dense with good contrast; (2) the speckle pattern must be visible throughout the test; (3) speckle sizes should be no less than 3–5 pixels, as dictated by the optical setup (i.e. camera resolution, lens and specimen stand-off distance); (4) intrinsic and extrinsic camera parameter calibration must be performed [25]. To measure the elastic deformation of each strip in the present study, speckle patterning was done manually using a toothbrush with alcohol-based ink to create random, fine speckles on the surface of the strip facing the camera. The DIC algorithm correlated the random speckle pattern in the images and tracked the displacement of the speckles to calculate and visualize strain.

**Fig. 3:**
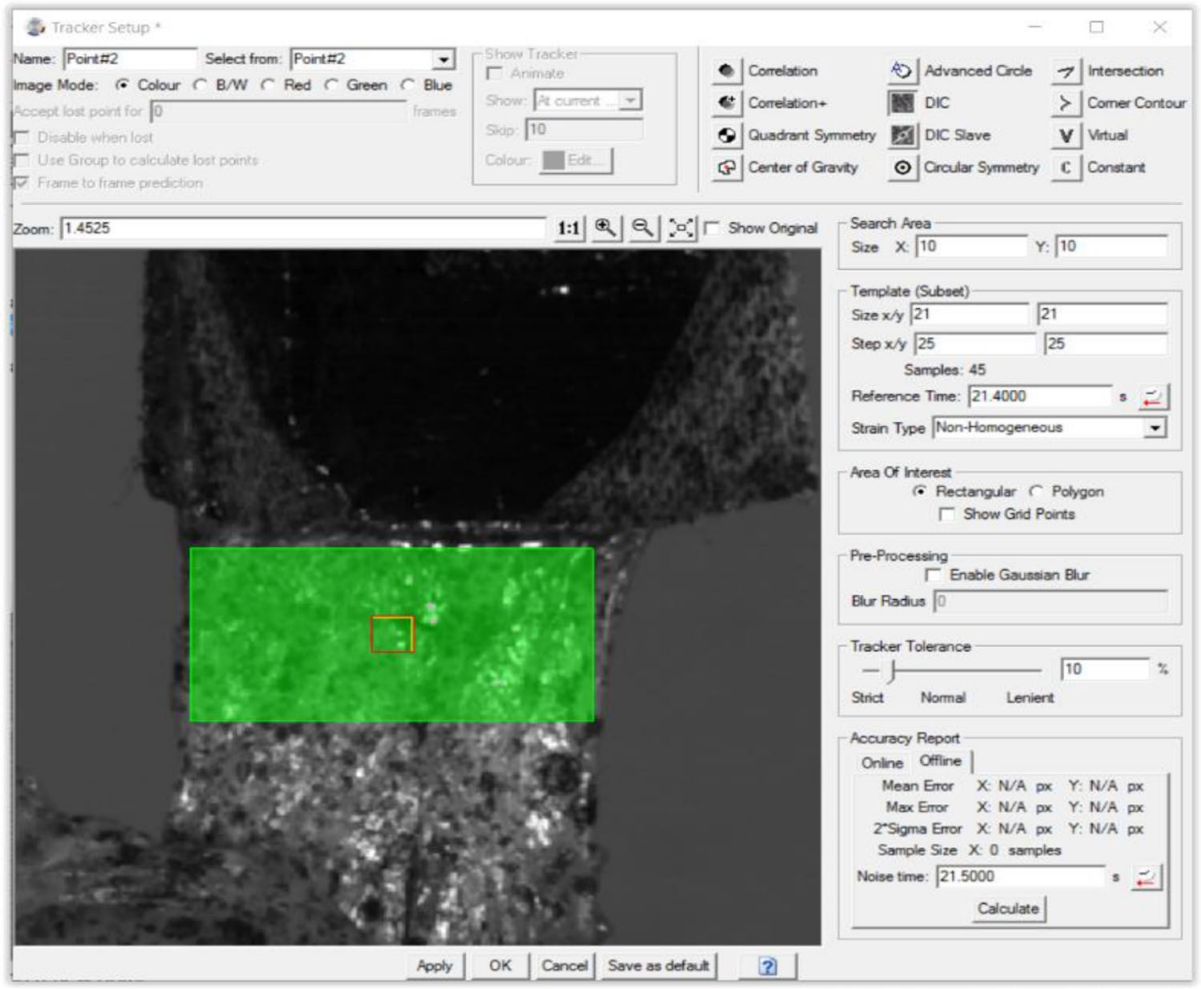
DIC tracker definition analysis

For each specimen, the capsular layer was tested before the tendinous layer and testing of supraspinatus, infraspinatus, and subscapularis was randomized. Once the specimen was fixed to the Instron and could be mechanically loaded, the cameras were activated and began documenting the strip displacement. A DIC facet size of 21 × 21-pixels was used with a 25-pixel step size (Fig. 3).

**Fig. 4:**
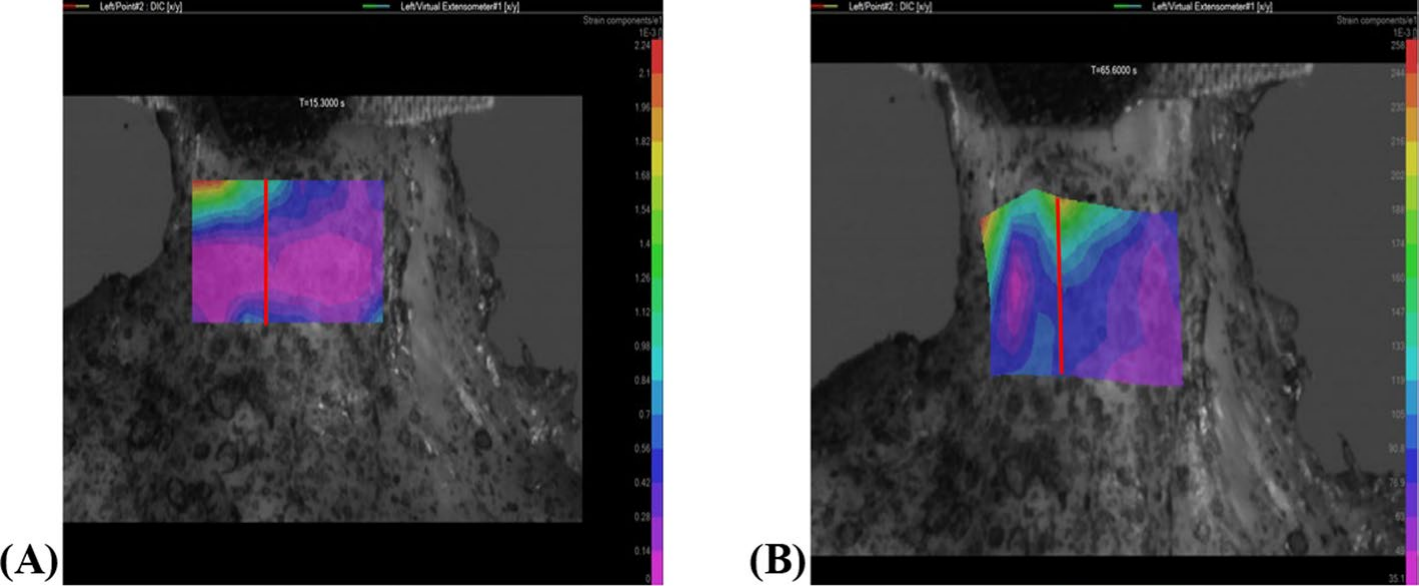
DIC for the tendinous layer of an infraspinatus strip where **A** shows the DIC strain field at 15.3 s and **B** DIC strain field at 65.6 s. The virtual extensometer is indicated in red

As the strip was mechanically loaded, calculated strains were mapped onto an area of interest on which the cameras focussed. In Fig. 4, a virtual extensometer is added in line with the clamp holding the strip and the load path, to obtain average engineering strain across the area of interest.

The capsular and tendinous portions of the supraspinatus, infraspinatus, and subscapularis of each of the samples were mechanically loaded to failure. These DIC results then underwent post-processing where uniaxial strain was calculated.

Elastic modulus, calculated from the slope of a stress–strain curve, has traditionally been calculated with Hooke’s Law $E = \sigma /\mathcal{E}$ when a linear homogeneous material is tested [26, 27]. However, the RC complex consists of soft tissue that is non-homogeneous, nonlinear, and anisotropic, therefore requiring a modified method for calculating elastic modulus [21]. Mallett and Arruda employed digital image correlation (DIC) on the anterior cruciate ligaments of bovine specimens to calculate full-field deformation, which was then used to construct stress/strain curves from tangent elastic moduli values obtained [21, 28]. When viewing these graphs, engineering stress is denoted as σ and conveys force applied (F) to the tissue divided by its cross-sectional area (A) as shown in Eq. (1) below, while strain $\mathcal{E}$ conveys the deformation a material undergoes. In Eq. (2), $\delta l$ represents the change in length of the specimen and* l* its original length:

1 $$\sigma = \frac{F}{A}$$

2 $$\varepsilon = \frac{\delta l}{l}$$

**Fig. 5:**
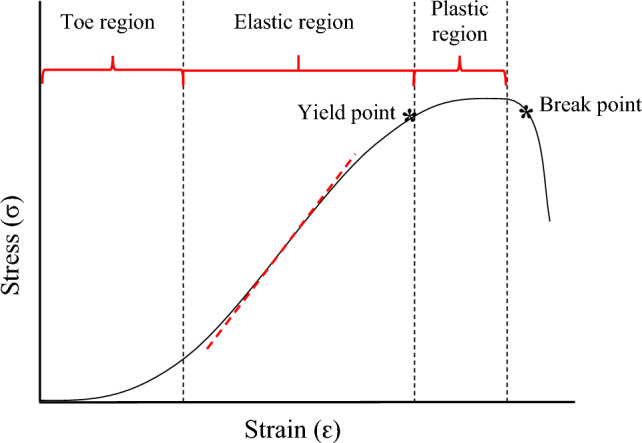
Graph demonstrating the regions, yield point and break point as they appear on a typical $\sigma /\varepsilon$ graph and a tangent line (red dash line) indicating where tangent elastic modulus information would typically be gathered

The toughness of tissue is measured by viewing the surface area underneath the $\sigma /\mathcal{E}$ curve, where larger surface areas imply that more energy will be required to break the material [29–31]. A typical $\sigma /\mathcal{E}$ data series for soft tissue is shown in Fig. 5. An initial toe region is observed where the material response is driven by elastin fibres, after which the elastic region mainly driven by collagen fibres is observed.

Engineering stress was calculated for each sample using Eq. 1. Elastic modulus was obtained from the tangent modulus, fitted to the point of maximum slope of the $\sigma /\mathcal{E}$ curve. This typically correlates with the linear–elastic portion (elastic region) of the specimen’s $\sigma /\mathcal{E}$ curve, where the collagen fibres are fully extended and the specimen is at its stiffest. Beyond this region fibre failures occur (plastic region), and the material stiffness decreases [32].

## Results

Reverse dissection of the RC muscles from their origins revealed that the supraspinatus, infraspinatus, and subscapularis insert across the humeral tuberosities as interdigitated tendinous and capsular sheets in accordance with previous literature [1, 2]. The thickness measurements of the test strips were recorded (prior to tensile loading) and are summarized in Table 1.

**Table 1:** Tendon thickness (in mm) measured prior to tensile loading

Specimen	Layer	SST	SSC	IST	ISC	SCT	SCC
001		5.20		**5.60**	2.10	4.70	3.20
002		2.80		3.70	1.80	4.50	3.20
003		4.60	**3.40**	4.00	2.60	**6.80**	3.40
004				4.20	**3.20**	3.80	2.10
005		3.75	1.00	4.00	1.50	4.60	2.40
006				4.35	2.20	3.50	2.20
007		**5.30**	2.80	4.60	2.80	6.30	3.00
008		4.75	3.00	4.10	1.10	4.10	**4.60**
**Average**		4.40	2.55	4.32	2.16	4.79	3.01
**SD**		0.96	1.06	0.58	0.69	1.17	0.81
**95% CI:**	**Lower**	3.39	0.86	3.83	1.58	3.81	2.33
	**Upper**	5.41	4.24	4.81	2.74	5.77	3.69

CI, confidence interval; ISC, infraspinatus capsular layer; IST, infraspinatus tendinous layer; SCC, subscapularis capsular layer; SCT, subscapularis tendinous layer; SD, standard deviation; SSC, supraspinatus capsular layer; SST, supraspinatus tendinous layer; Bold print values represent thickest recording of that layer recorded throughout testing

Once the thicknesses of all strips had been recorded, they were subjected to tensile loading, and Table 2 summarizes the yield points per RC section for each sample provided as stress (kPa); strain (unitless) values.

**Table 2:** Yield points recorded as strain; stress [kPa] obtained during tensile loading

Sample	SST	SSC	IST	ISC	SCT	SCC
001	0.079; 3281.9		0.133; 6740.0	0.225; 6105.1	0.262; 6692.8	0.191; 8476.6
002	0.136; 1449.8		0.178; 3809.6	0.210; 2855.5	0.118; 3234.4	0.227; 4606.9
003	0.088; 4813.0	0.092; 932.2	0.187; 4670.4	0.076; 3195.6	0.044; 2107.2	0.234; 3745.1
004			0.233; 6371.0	0.118; 3562.9	0.085; 6483.8	0.154; 5004.4
005	0.107; 5067.7	0.177; 6339.8	0.094; 3171.9	0.114; 4320.7	0.100; 3383.2	0.157; 5218.8
006			0.052; 6150.1	0.078; 2800.6	0.075; 4702.4	0.202; 8179.4
007	0.070; 2504.9	0.074; 1923.5	0.231; 4914.5	0.118; 691.8	0.139; 3390.9	0.150; 2851.7
008		0.067; 3267.0	0.090; 4737.9	0.111; 4509.9	0.198; 7962.9	0.140; 1979.2
**Mean**	0.096; 3423.5	0.103; 3115.6	0.150; 5070.7	0.131; 3505.3	0.128; 4744.7	0.182; 5007.8
**SD**	0.026; 1532.4	0.051; 2352.8	0.072; 1186.5	0.056; 1575.1	0.071; 2073.7	0.037; 2321.1
**CIs: Upper**	0.129; 5326.2	0.183; 6859.4	0.206; 6125.0	0.178; 4822.1	0.187; 6478.4	0.213; 6948.2
**CIs: Lower**	0.063; 1520.7	0.022; -628.2	0.093; 4016.3	0.084; 2188.4	0.068; 3011.0	0.151; 3067.3

CI, 95% confidence interval; ISC, infraspinatus capsular layer; IST, infraspinatus tendinous layer; SCC, subscapularis capsular layer; SCT, subscapularis tendinous layer; SD, standard deviation; SSC, supraspinatus capsular layer; SST, supraspinatus tendinous layer

The tendinous layers for supraspinatus and infraspinatus demonstrated higher tangent moduli, and on average higher tensile strengths compared to their capsular counterparts. Subscapularis reacted contradictory to this, where the tendinous layers frequently achieved lower tensile strengths.

Table 3 presents the elastic modulus recorded per RC section for each sample. Bolded values indicate the highest elastic modulus value obtained for the capsular and tendinous layers for each the supraspinatus, infraspinatus, and subscapularis.

**Table 3:** Tangent elastic modulus (MPa) records obtained from graphs generated during DIC post-processing

Sample	SST	SSC	IST	ISC	SCT	SCC
001	68.7		82.7	31.3	32.6	**76.6**
002	20.9		52.9	21.3	79.5	24.6
003	76.0	11.1	38.8	**42.8**	55.5	22.6
004			30.5	36.6	**84.8**	44.3
005	**108.4**	38.9	47.3	41.2	54.7	57.5
006			**203.8**	38.3	45.1	60.8
007	36.3	26.2	22.9	5.9	78.1	22.8
008		**39.8**	57.5	42.4	46.7	22.8
**Mean**	62.1	29.0	67.1	32.5	59.6	41.5
**SD**	34.4	13.5	58.2	12.9	19.0	21.4
**CI Lower**	19.3	7.6	18.4	21.7	43.8	23.6
**CI Upper**	104.8	50.4	115.7	43.3	75.5	59.4

CI, 95% confidence interval; ISC, infraspinatus capsular layer; IST, infraspinatus tendinous layer; SCC, subscapularis capsular layer; SCT, subscapularis tendinous layer; SD, standard deviation; SSC, supraspinatus capsular layer; SST, supraspinatus tendinous layer; bold print values represent highest elastic modulus recording of that section/layer recorded throughout testing

**Fig. 6:**
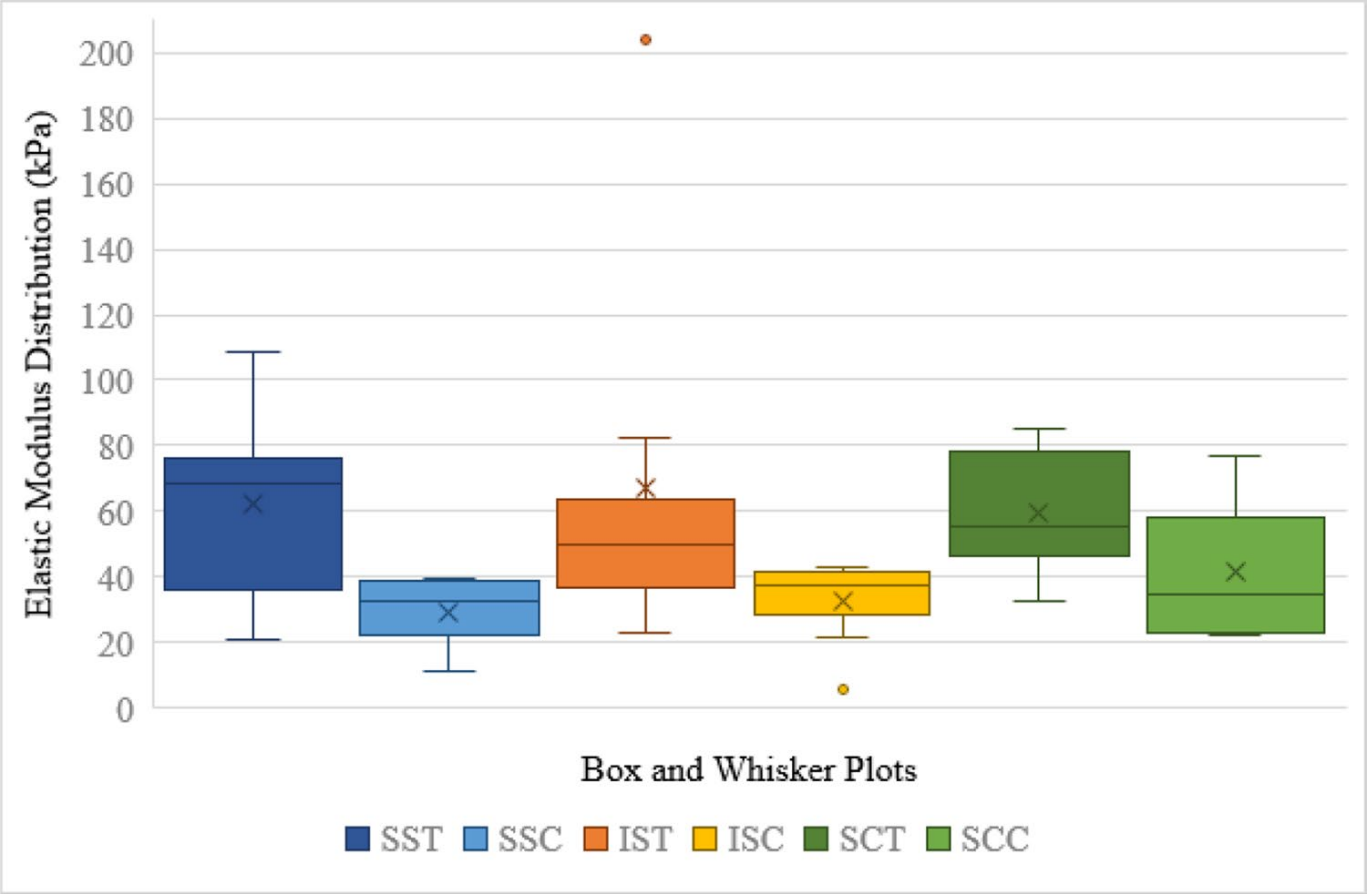
Box and whisker plots for tendinous and capsular layers of supraspinatus (SST and SSC, respectively), infraspinatus (IST and ISC, respectively), and subscapularis (SCT and SCC, respectively)

Figure 6 depicts the range of the tangent elastic modulus data obtained for each RC segment group where outlier values are indicated for IST at 203.8 MPa and ISC at 5.9 MPa. From this figure, it can clearly be seen that the tendinous layer for each of the RC segments displayed with greater elastic modulus averages than their respective capsular counterparts. More data dispersion around the mean can also be seen for SST and IST when compared to SSC and ISC.

Table 4 summarizes the data distribution displayed in Fig. 6 for the tendinous and capsular layers of supraspinatus, infraspinatus, and subscapularis.

**Table 4:** Elastic modulus descriptive distribution values

	SST	SSC	IST	ISC	SCT	SCC
Min	20.9	11.1	22.9	5.9	32.6	22.6
Q1	36.3	22.4	36.7	28.8	46.3	22.8
Median	68.7	32.6	50.1	37.4	55.1	34.5
Q3	76.0	39.1	63.8	41.5	78.5	58.3
Max	108.4	39.8	203.8	42.8	84.8	76.6
Sample size (n)	5	4	8	8	8	8
Range	87.5	28.7	180.9	36.9	52.2	54.0
IQR	39.7	16.7	27.1	12.7	32.2	35.5
Lower Limit	-23.3	-2.6	-3.9	9.8	-1.9	-30.5
Upper Limit	135.6	64.2	104.4	60.6	126.7	111.6

ISC, infraspinatus capsular layer; IST, infraspinatus tendinous layer; IQR, interquartile range; SCC, subscapularis capsular layer SCT, subscapularis tendinous layer; SSC, supraspinatus capsular layer; SST, supraspinatus tendinous layer

## Discussion

The findings of the present study demonstrated that the capsular layers generally allowed for greater material deformation before breaking, despite not being able to withstand as high stresses as the tendinous layers. The smaller range in stress yield for the subscapularis capsular portion in the results signifies that the capsular portion of subscapularis is stiffer than the capsular portions of supraspinatus and infraspinatus. The capsular portions of supraspinatus and infraspinatus are therefore able to stretch further under the same load than the capsular portion of subscapularis.

Among the first to study biomechanical properties of the RC complex were Nakajima and co-authors [33]. The authors found elastic differences between two macroscopic layers of the supraspinatus as partitioned in their study by dividing the muscle into two equally thick sheets to represent the articular and bursal layers. The articular portion displayed a slightly greater elastic modulus average (of 8.2 MPa ± 0.2 M Pa) when compared to the overlying bursal portion (7.2 MPa ± 0.3 MPa). They also noted that RC tear patterns did not vary with age within their sample population (40 shoulders from individuals aged 28–79 years). When comparing the results from the present study with those observed by Nakajima et al., notably higher average elastic modulus values were obtained for both the tendinous and capsular portions. The difference in elastic modulus findings between the two layers of a given muscle was also much larger than reported by Nakajima et al. [33]. A reason for this could be the method of partitioning the muscles in the present study. Considering the histological division of the RC muscles and their macroscopic translation, the present study sectioned the muscles into capsular and tendinous parts through the fascial plane towards the insertion, instead of dividing them equally as described by Nakajima et al. which may not have represented the total elastic capacity of the capsular and tendinous layers in their study. Additionally, the use of DIC allowed for a more detailed investigation of the deformation within the RC components compared to previously available techniques.

As previously documented, the present study likewise found that the supraspinatus, infraspinatus, and subscapularis of the RC unit originate on the scapula and extend towards the proximal aspect of the humerus as interconnected, interdigitating superficial tendinous fibres interdigitating with the deeper capsular layer [1, 2, 34]. The RC unit inserts across the lesser and greater tuberosities of the humerus as two wide and overlapping layers which are the main stabilizing factors of the very mobile glenohumeral joint [35]. Approximately 15 mm from the insertion onto the humerus the supraspinatus, infraspinatus, and subscapularis are indistinguishable from one another, save for following the course of their muscular origins into the capsular–tendinous junction. The capsular layer thus, together with the tendinous layer, serves as the insertional footprint of the RC due to this interdigitation. The combined insertion of these RC muscles onto the humeral tuberosities has been histologically described as a fibrocartilaginous zone, a tendon–bone interface that has been documented to be more resilient to strain considering its structure and composition [1, 2, 36–38]. In the present study, this did not factor into the biomechanical findings considering the tear zones noted were not localized at this interface, but instead the DIC employed focussed on a pre-defined area to observe the elastic deformation and eventual failure of the tissue.

In the present study, yield points indicate the approximate point past which the RC section became plastically deformed and the elasticity readings past this point were observably lower. The yield point was observed where strain increased more rapidly than stress which brought on permanent deformation in the tissue. The yield point is important in biomedical technology as it is the value which materials used in reparative operations are compared to, ensuring they have a greater yield strength than the affected region, to withstand forces that will act on this region postoperatively [39, 40].

Studies on the more frequent occurrence of articular compared to bursal-sided tears suggest this may be attributed to the relatively disorganized interwoven collagen fibrils seen in the deeper histologic layers compared to the superficial tendinous layer consisting of thicker, more organized tendon fibres [3, 41]. When considering the findings from the present study, the capsular layers of the RC sections generally yielded lower elastic modulus readings (n = 14/19) than their tendinous counterparts of the same shoulder. It is known that a higher elastic modulus (which corresponds to a steeper $\sigma /\mathcal{E}$ slope) demonstrates a stiffer material [42]. Elastic modulus findings can be interpreted as the same response from materials that are both strong (in the present study the tendinous more so than capsular layer) and ductile. Thus, as the RC tendinous and capsular layers are exposed to chronic loading that compromises inherent tissue characteristics, tension on the stretched fibres leads to stiffening. RC tearing can therefore be translated, opposed to the original tissue properties, as stiffened fibres that are more susceptible to failure. Decreased load resistance and elastic modulus outputs obtained for the capsular layers in the present study, as well as consideration of the tissue composition, could explain more commonly observed capsular sided tears as opposed to tears found within the tendinous layers.

Figure 1 highlights how the tendinous layers of the muscles analysed achieved greater elastic modulus averages than their capsular counterparts. Although the distribution around the median was wider for the tendinous layers, the capsular layers were all within the 30–40 range of elastic modulus distribution. This could imply that the capsular layers respond more similarly as a whole to load bearing than their tendinous counterparts, which are more individually affected and able to resist loads to different capacities.

### Limitations

The following limitations were encountered: sample size, equipment, soft tissue characteristics. The small sample size is attributed to the high costs associated with fresh tissue samples as well as the extensive time needed to test each specimen. However, despite the small sample size, the elastic differences between the two macroscopic layers were emphasized and shown to be significantly greater than previously reported, as reflected in the results. The clamp used to grip the rotator cuff strips was narrower than the strips themselves which resulted in uneven load distribution across the strip being tested. This, however, did not affect the ultimate yield point of RC section as the region of interest was narrowed to the point at which failure occurred. As the study focus pertained to testing the separate layers of the rotator cuff, therefore leaving only short sections of the samples attached to the humerus, the sample aspect ratio was not as would be typically expected for the purpose of the mechanical characterization of biological materials. The inherent properties of the soft tissue also provided testing difficulties—the moist, fresh tissue proved difficult to clamp for tensile loading. Measures were implemented prior to loading to secure the strips within fine-toothed clamps layers with sandpaper. However, humeral shaft pull-out and some strip slippage occurred necessitating second tests. Unlike simple pull-out and strip slippage that occurred at the very start of the tests as soon as the base plate was lowered, occasionally a strip would slip at greater loads. In these cases, where second loading tests needed to be conducted, the strip was noted to perform worse on the second test. This may have been due to micro-failure in the tissue, and to avoid bias in the results, these tests were excluded entirely from the findings. In this work, speckle patterns could only be applied to the tissue after placing the specimens in the Instron setup. Several options were evaluated to apply speckle patterns to the specimens including airbrushing with a speckle pattern template, ink stamps, and temporary tattoo paper speckle pattern transfer. For this setup, applying the speckle pattern with a toothbrush was the most reliable and practical approach. This did, however, prevent control over speckle dot sizes, which affected the DIC data quality. A focus of future research would be finding better approaches to apply speckle patterns to fresh tissue specimens, in order to maximize DIC data quality.

## Conclusion

Past research on the RC revealed small biomechanical differences between the capsular and tendinous layers. The methodology of distinguishing these layers and recording their tensile properties may not have captured the true elastic variations between the layers, however. The present research, employing modern technologies and sectioning the RC muscles through their histologically distinguished layers, revealed much larger elastic differences between the capsular and tendinous portions than previously recorded. This thus sheds additional light on differences between the macroscopically distinct layers not only in terms of composition but also biomechanical elastic qualities. The tendinous portions proved stronger than the capsular portions and could therefore be expected to endure under greater loads compared to the capsular layer. If these layers are not repaired individually, significant biomechanical imbalance could be experienced by patients having undergone RC tear surgery as repairing them as one could significantly impact the ductility of the involved segment. Knowledge of the biomechanically distinct functions of the muscles, as well as the inherent elastic differences between the macroscopic layers, could lead to developing improved operative protocols for repairing tears found in one or both macroscopically distinct RC layers. This information along with the elastic modulus outputs for the understudied infraspinatus and subscapularis muscles may additionally prove useful for future finite element studies, providing more context on the load bearing mechanics of these unique layers.

## Data Availability

No datasets were generated or analysed during the current study.

## Abbreviations

A: Cross-sectional area
A/L: Anterolateral
A/M: Anteromedial
CI: Confidence interval
DIC: Digital image correlation
Ε: Deformation specimen has undergone/strain
F: Force
I/D: Inferior/distal
ISC: Infraspinatus capsular layer
IST: Infraspinatus tendinous layer
kPa: Kilopascals
*l*: Original length
MPa: Megapascals
MTS: Material test systems
RC: Rotator cuff
σ: Stress
SCC: Subscapularis capsular layer
SCT: Subscapularis tendinous layer
*δl*: Change in length
SD: Standard deviation
S/P: Superior/proximal
SSC: Supraspinatus capsular layer
SST: Supraspinatus tendinous layer

## Declarations

The following sections detail any and all declarations relevant to the study.
